# Translational control of recombinant human acetylcholinesterase accumulation in plants

**DOI:** 10.1186/1472-6750-7-27

**Published:** 2007-05-30

**Authors:** Brian C Geyer, Samuel P Fletcher, Tagan A Griffin, Michael J Lopker, Hermona Soreq, Tsafrir S Mor

**Affiliations:** 1 The School of Life Sciences and Biodesign Institute, P.O. Box 874501, Arizona State University, Tempe, AZ 85287-4501, USA; 2 The Dept. of Biological Chemistry, The Institute of Life Sciences, The Hebrew University of Jerusalem, Jerusalem 91904, Israel

## Abstract

**Background:**

Codon usage differences are known to regulate the levels of gene expression in a species-specific manner, with the primary factors often cited to be mRNA processing and accumulation. We have challenged this conclusion by expressing the human acetylcholinesterase coding sequence in transgenic plants in its native GC-rich sequence and compared to a matched sequence with (dicotyledonous) plant-optimized codon usage and a lower GC content.

**Results:**

We demonstrate a 5 to 10 fold increase in accumulation levels of the "synaptic" splice variant of human acetylcholinesterase in *Nicotiana benthamiana *plants expressing the optimized gene as compared to the native human sequence. Both transient expression assays and stable transformants demonstrated conspicuously increased accumulation levels. Importantly, we find that the increase is not a result of increased levels of acetylcholinesterase mRNA, but rather its facilitated translation, possibly due to the reduced energy required to unfold the sequence-optimized mRNA.

**Conclusion:**

Our findings demonstrate that codon usage differences may regulate gene expression at different levels and anticipate translational control of acetylcholinesterase gene expression in its native mammalian host as well.

## Background

Different organisms preferentially use a different subset of the synonymous codons specifying a certain amino acid. In fact, codon usage is emerging as an important regulatory factor, and codon usage bias can be detected not only between organisms [1], but also in the same organism between differentially expressed genes [2], in different tissues [3] and developmental stages [4]. A correlation was found in some cases, most notably among prokaryotes and in some metazoans (e.g. in dipterans and in nematodes), between the codon usage and the abundance of the cognate tRNAs, indicating that in these cases at least codon usage is probably governed by evolutionary selective forces [5-8]. However, "translational selection" may not be the only adaptive process shaping codon usage, which can be biased due to interactions not (directly) involving codon-anticodon interactions such as splice-site recognition [9] and mRNA turnover [10]. Particularly, while the available genome-scale analyses done for several plant species demonstrate that codon bias in plants is adaptive [11,12], heterologous gene expression studies in plants led to a tentative conclusion that translation efficiency of the foreign genes is often considered secondary to mRNA processing and stability as a determinant for expressivity [13].

For many reasons, acetylcholinesterase, (AChE) is a useful model for a comparative study addressing the issue of codon bias and translation efficiency, as well for post-translational events [14,15]. Vertebrate genes encoding the enzyme from several sources were expressed in a wide variety of systems such as bacteria [16], yeast [17], mammalian cell cultures [18], transgenic mice [19] and more recently in plants [20,21]. The enzyme is known for its role in the termination of synaptic transmission by hydrolyzing acetylcholine, but during the past decade evidence has been gathered indicating its critical role in various processes such as development, stress responses, innate immunity, and bioscavenging [14]. This complex array of functions is enabled through the equally complex molecular biology of the *ACHE *gene: the intricate control of its transcription, alternative splicing and translation and the equally intricate post-translational events governing its function such as subcellular targeting, glycosylation, proteolytic processing, membrane anchoring and protein-protein interactions [14]. Interestingly, non-cholinergic functions of AChE are underscored by identification of AChE-like activity and genes potentially encoding the enzyme in several plant species [21-23].

Original reports from our laboratory indicated that plants can express this complex human enzyme. However, yields of plant-expressed AChE were modest because of limitations in the prototype expression construct and host plant [20,21]. Here we demonstrate that AChE accumulation in plants is limited at the translation stage and that the enzyme levels can be dramatically increased as compared to our previously reported results, by conforming its codon usage and GC content to that of highly expressed plant genes. The optimized sequence results in increased translatability of the mRNA without affecting its accumulation or processing.

## Results

### Design and cloning of the plant-expression optimized *oACHE-S *gene

**Figure 1:**
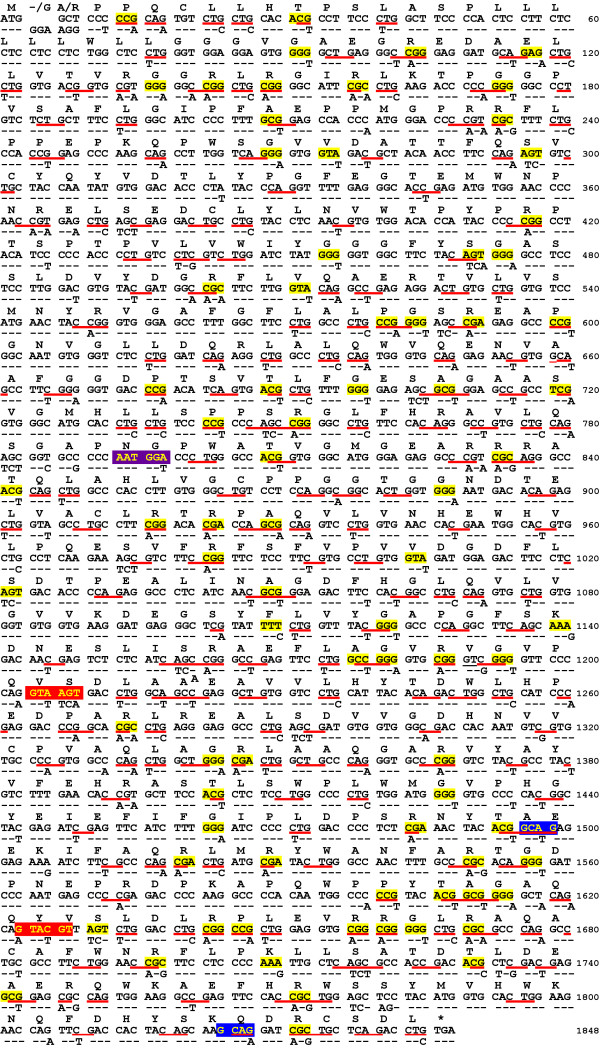
**Sequence optimization of the *hACHE-S *gene**. Shown are the native (cDNA) human gene sequence (upper) and the optimized gene (lower). Nucleotide changes are shown, whereas unmodified nucleotides are represented by dashes. Above the two compared DNA sequences is the predicted amino-acid sequence of the *AChE-S *protein. Underline: potential methylation sites. Yellow box: infrequent plant codons. Red box: potential 5'-intron splice sites. Blue box: potential downstream 3'-intron splice sites. Purple box: a potential polyadenylation positional element. Please note that the only amino-acid deviations from the human cDNA are an R2A change in the human sequence and insertion of a G residue on this position in the plant-optimized sequence. These changes were introduced to facilitate cloning into the plant expression vectors used in this study.

**Figure 2:**
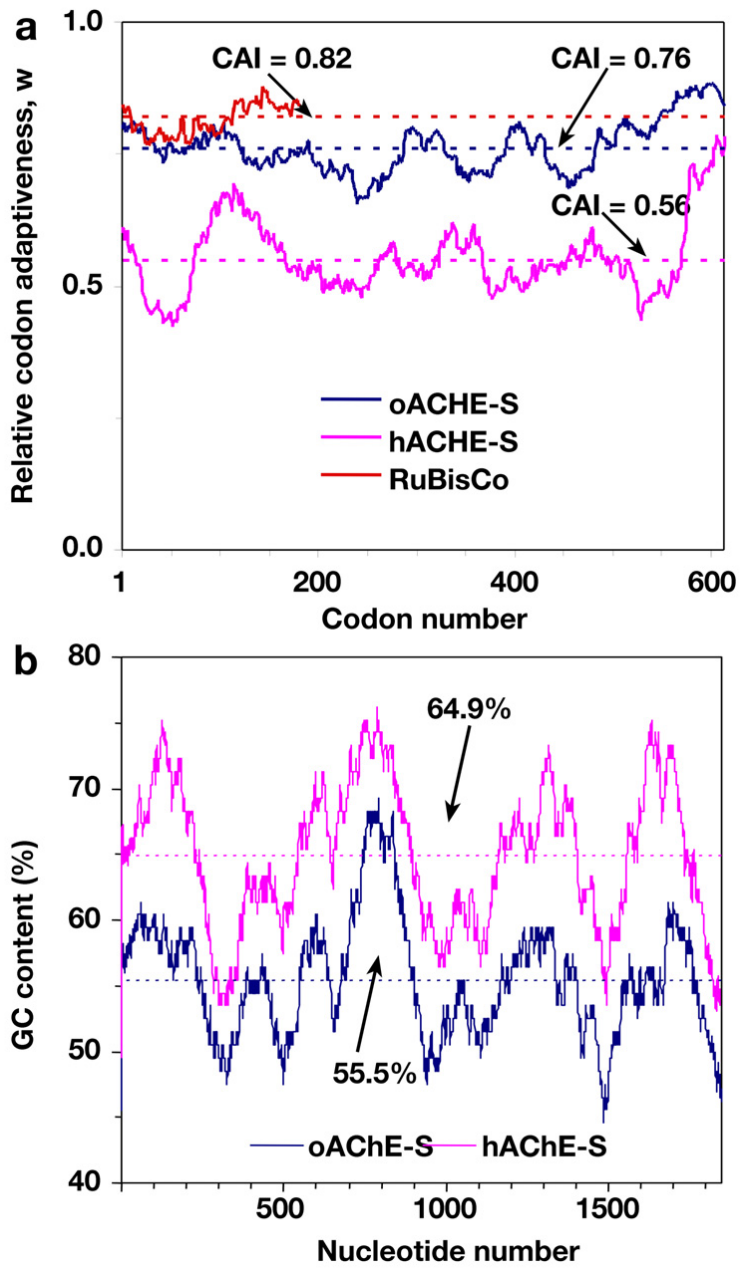
**Comparison of the codon usage and GC content of *hACHE-S *and *oACHE-S***. **(A) **Codon usage, expressed as a moving (window = 50 codons) geometric mean of the relative codon adaptiveness (*w*) was scored for *hACHE-S *(pink), *oACHE-S *(blue), and the highly-expressed plant gene *rbcS *(red). The CAI values of each (*w *averaged over the entire gene) are plotted as broken lines. Note that optimization increased the CAI value of the *ACHE *gene, bringing it close to the CAI value of the *rbcS *gene, which is even for this highly expressed gene lower than the theoretical maximal value of 1. **(B) **The GC content is scored for 100-base segments centering at each of the indicated bases of the *hACHE *and *oACHE *mRNA (taking into account the common TEV leader). Broken lines – the GC content of the entire coding regions.

More than a third of the codons of the human *ACHE *gene (*hACHE-S*) are infrequently utilized in dicotyledonous plants (*i.e*. as defined in the Methods section, they have relative synonymous codon usage, RSCU, values of less than 0.8, Fig. 1, Fig. 2, Table 1). Furthermore, a non-canonical near-upstream element of a plant polyadenylation signal [24,25] (Fig. 2, Table 1) and two plant 5'-intron splice signals with downstream 3'-splice signals were identified in *hACHES *[26,27] (Fig. 2, Table 1). In addition, potential methylation signals associated with transcriptional silencing (CG and CNG) [28] are abundant in the gene, reflecting its high GC content (65%, Fig. 1, Fig. 2, Table 1). Interestingly, the GC content in mid-exonic regions is even higher, with peaks exceeding 75% (Fig. 2). These regions are separated by deep troughs with much lower GC content (<50%). In designing a plant-expression optimized version of the gene, we took measures to correct for these potential problems. Thus, the rare codons present in *hACHE-S *were changed in the synthetic plant-expression optimized *ACHE *gene (*oACHES*) to more frequently used alternatives so that the codon adaptiveness index (CAI, for definition see the Methods section) would match that of the most abundant nuclear-encoded plant protein – the small subunit of ribulose bisphosphate carboxylase (RuBisCO, Fig. 2 and Table 1). Furthermore, the RNA processing signals were abolished, most of the potential methylation signals were eliminated, and the overall GC content was reduced to 55%, much closer to the typical plant gene (Fig. 2, Table 1). In particular, most of the GC content peaks were considerably dampened. Please note that our optimization strategy did not eliminate all infrequently used codons, giving preference to the elimination of all other deleterious sequences. However, the content of such (evenly distributed) codons was reduced to about 20% (similar to the case of the RuBisCO small subunit, Fig. 2, Table 1). When cloning *hACHE-S *and *oACHE-S *into plant expression vectors, two minor changes in the amino acid sequence of the encoded proteins were required due to the need to introduce an *Nco*I site to facilitate the cloning (Fig. 1). These deviations from the human sequence (R2A in *hACHE-S *and insertion of G between M1 and R2 in *oACHE-S*) are both in the cleavable signal peptide directing the protein to the secretory pathway.

**Table 1:** Optimized molecular features.

**Molecular Feature**	**hACHE-S**	**oACHE-S**
Codon usage^a^		
Uncommon	221 (36%)	89 (14%)
Other	393 (64%)	526 (86%))
Potential methylation sites^b^		
CG	110	0
CNG	192	82
Polyadenylation signal^c^		
AATGGA	1	0
5'-intron splice signals^d^		
GTAAGT	1	0
GTACGT	1	0
GC content	64.9%	55.5%

### Optimization of transgene enhances *AChE *accumulation in plants

**Figure 3:**
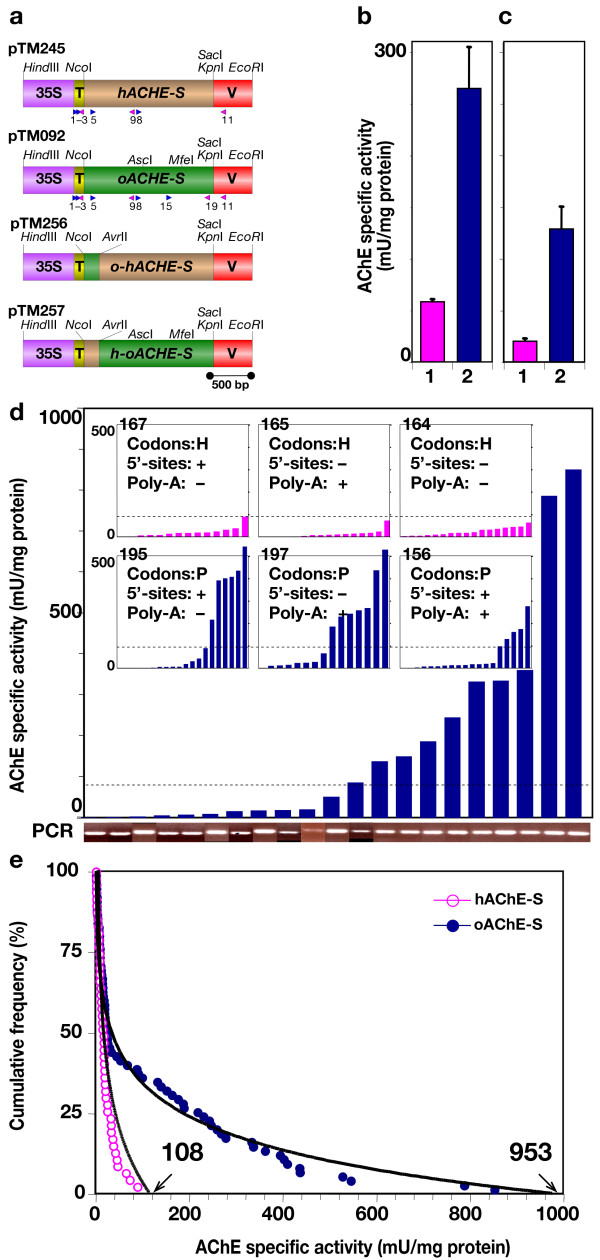
**Optimization of transgene enhances AChE accumulation in plants**. (**A**) Plant expression cassettes used in this study. pTM245 driving the expression of *hACHE-S*. pTM092 driving the expression of *oACHE-S*. pTM256 driving mosaic *o-hAChE-S*. pTM257 driving the expression of mosaic *h-oAChE-S*. 35S, CaMV 35S promoter; T, TEV leader; V, VSP terminator. Arrow heads, positions of oligonucleotides used in this for making probes, genomic PCR and RT-PCR (numbers refer to Table 2). (**B) **Transient expression assays in *A. tumefaciens*-infiltrated leaves of *N. benthamiana *plants. 1, *hACHE-S*; 2, *oACHES*. Averages of 6 replicates (means ± SEM) are shown. (**C**) *N. benthamiana *plants were stably transformed to express (1) *hACHE-S *or (2) *oACHE-S*. Individual lines were screened at least 2 times. Averages of all lines (presented in Panel D) with detectible levels of AChE (means ± SEM) are shown. (**D**) AChE accumulation levels in individual genomic-PCR positive plant lines expressing *oACHE-S*. Top inserts: AChE accumulation levels in individual plant lines expressing variants of *hACHE-S*: pTM167, pTM165 and pTM164. Bottom inserts: AChE accumulation levels in individual plant lines expressing variants of *oACHE-S*: pTM195, pTM197 and pTM156. Broken lines illustrate the maximal level of expression obtained with any of the *hACHE-S *lines. DNA fragments were PCR amplified from total DNA isolated from the indicated plant lines with *oACHE *specific primers (oTM126 and oTM047) and were resolved by agarose gel electrophoresis (denoted PCR). (**E**) Cumulative frequency distribution of the AChE expression data of lines shown in Panel D (high to low) can be fitted with the following logarithmic regression equations: *hACHE-S*, y = -22.967Ln (x) + 107.63 (r^2 ^= 0.90); *oACHE-S*: y = -15.013Ln (x) + 102.99 (r^2^= 0.98).

Binary expression vectors pTM245 and pTM092 encode *hACHE-S *and *oACHE-S*, respectively (Fig. 3A). We compared their expression in *N. benthamiana *leaves by *Agrobacterium*-mediated transient assay. In the *hACHE-S-*infiltrated leaves AChE-S accumulated to 58 ± 3 mU/mg protein, whereas the optimized sequence was expressed at 264 ± 40 mU/mg protein (mean ± SEM, Fig. 3B). Thus, the accumulation level of the enzyme with the plant-expression optimized gene was about 5 fold higher than obtained with the human sequence.

In optimizing the human *ACHE-S *gene sequence, we took a preemptive approach: modifying many molecular features that might have otherwise proven deleterious to the expression of the transgene or to the accumulation of its product. Thus we have modified key signals leading to transcriptional silencing (methylation), aberrant transcript processing (premature polyadenylation and unwarranted splicing) and translation attenuation (codon usage). It was therefore of interest to explore which of these changes contributed to the dramatic improvement in AChE accumulation observed with the optimized construct. To this end we removed the two 5'-splice sites, the potential poly-A site or all from the human sequence creating constructs pTM167, pTM165 and pTM164 respectively. Conversely, we reintroduced the same sites into the plant-expression optimized sequence, creating pTM195, pTM197 and pTM156 respectively. These new AChE-encoding plant expression constructs, alongside the pTM092 and pTM049 were introduced into *N. benthamiana *by *Agrobacterium-*mediated gene transfer (Fig. 3C–E).

Because of the integration position effect, transformants harboring the transgenes exhibited a range of recombinant gene expression levels, manifested in the distribution of AChE activities observed (Fig. 3D). Only kanamycin-resistant plants that had detectable (non-zero) AChE activity were considered for the following analyses. Accumulation of AChE activity significantly differed (*P *< 0.0001) between plants expressing variants of *hACHES *and plants expressing *oACHES *(and variants thereof). The highest AChE activity level obtained among the *hACHES *plants was 89 mU/mg protein, with a mean of 20 ± 3 mU/mg protein (n = 47, Fig. 3C). These results are somewhat higher but comparable to results previously described by us regarding the expression of the non-optimized sequence encoding the catalytic core of the enzyme in tomato plants [20]. In sharp contrast, the highest specific activity level demonstrated for *oACHES *plants was 852 ± 28 mU/mg protein and the observed mean was 129 ± 22 mU/mg protein (n = 75, Fig. 3C).

The distribution of AChE accumulation data (Fig. 3E) is to a good approximation lognormal, and can be fitted to a logarithmic function, which allows one to predict the theoretical maximal level of AChE accumulation that may be obtained with each of the construct families. These values are in a good agreement with the actual observed maximal values, being about 108 and 953 mU/mg protein for the human and optimized constructs, respectively (Fig. 3E). It therefore appears unlikely that by screening additional transformants we could have identified *hACHES *lines that would accumulate significantly higher levels of AChE, than those we report. We conclude that expressing *oACHES *constructs leads to a 10-fold increase in AChE accumulation as compared to the *hACHES *constructs, and that the enhancement is due to the sequence optimization and not due to insufficient transformant pool size.

### *hACHE-S *and *oACHE-S *transcripts accumulate to similar levels in transgenic plants

Our analysis of the human *ACHE-S *gene shows it contains several plant RNA processing signals that theoretically could lead to the production of truncated, presumably inactive, AChE. However, removing these sequences in *hACHE-S *(pTM167, pTM165 and pTM164) or their reintroduction into the *oACHES *sequence (pTM195, pTM197 and pTM156) did not have a considerable effect on the enzyme accumulation levels (Fig. 3D). In contrast to the highly significant difference between the two construct families, no significant differences were found among plants expressing variants of the *hACHES *sequence (*P *> 0.12) or the one expressing variants of the *oACHES *sequence (*P *> 0.07). This implies that the three potentially deleterious sequences (the two 5'-intron splice sites and the polyadenylation sequence) do not play a major role in this case.

**Figure 4:**
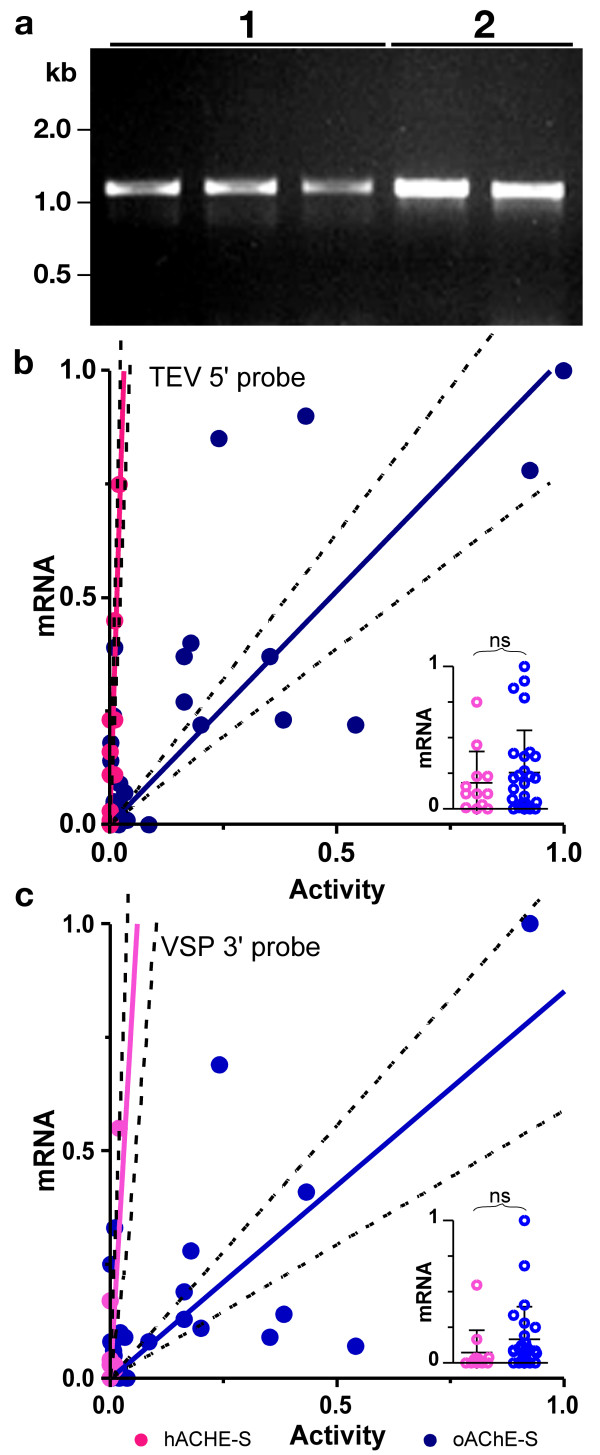
**Full-length *hACHE-S *and *oACHE-S *transcripts accumulate to similar levels**. (**A**) RT-PCR amplification of *ACHE *transcript region potentially subjected to aberrant splicing. cDNA prepared from three lines of *hACHE-S-R177W *plants (1) and two lines of *oACHE-S *plants (2) were subjected to PCR amplification using the primers oTM089 and oTM049 and then resolved by agarose gel electrophoresis. Primers were selected to amplify the region potentially subjected to alternative splicing. Unspliced transcripts are expected to yield 1.1 kb fragment. If either of the two potential 5'- and 3'-intron splice sites were extensively utilized in plants harboring *hACHE-S*, shorter fragments of ~0.9 kb and ~0.5 kb are expected to be preferentially amplified. However, the full-length species clearly dominates. (**B-C**) Correlation between *ACHE *transcript accumulation and AChE enzyme activity. Transcripts levels of optimized (pTM092) and non-optimized (pTM050) measured by quantitative real time RT-PCR and were scored relative to 18S rRNA. Probes were designed for the two common regions of the two types of *ACHE *transcripts – (**B**) the 5'-UTR (TEV leader) and (**C**) the 3'-UTR (VSP terminator, upstream of the poly-A tail). Data obtained for multiple replicates was averaged and correlated with the respective AChE activity (normalized to the maximum level). When probed by the 5'-UTR probe, the correlation was highly significant with r^2 ^= 0.72 (*P *< 0.0001) for the non-optimized sequence and r^2 ^= 0.61 (*P *= 0.0005) for the optimized sequence. When probed by the 3'-UTR probe, the correlation was significant for the non-optimized sequence with r^2 ^= 0.48 (*P *= 0.0122) and highly significant with r^2 ^= 0.49 (*P *< 0.0001) for the optimized sequence. Inserts show scatter plots demonstrating the distribution of transcript levels in plants expressing *hACHE-S *and *oACHE-S *constructs. Each plant is depicted by a symbol and the mean ± S.D. are plotted. The groups are not statistically different by an unpaired t test (5'-UTR probe: *P *> *0.45*; 3'-UTR probe: *P *> *0.22*).

To corroborate this conclusion, we amplified by PCR cDNA derived from either pTM050 (harboring *hACHE-S *with a single base change) or pTM092 (*oACHE-S*) lines using primers specific to the region encompassing the potential introns. In both cases the only fragments amplified corresponded to the correct, full length, sequence (Fig. 4A). Therefore, it seems unlikely that spurious splicing events are responsible for the poor performance of the *hACHE-S *construct. Similarly, an RNA blot analysis using probes specific for the human vs. plant-expression optimized sequences, demonstrated single, identically sized transcripts in both *hACHE-S *and *oACHE-S *of the predicted length (data not shown), abating the probability that premature polyadenylation was limiting *hACHE-S *expression as compared to *oACHE-S*.

Transcript accumulation levels and integrity were further assayed by quantitative real-time PCR analysis using primers and probes to either the 5'-UTR (TEV leader sequence) or to the 3'-UTR (VSP terminator), common to both *hACHE-S *and *oACHE-S*, and were normalized to the co-determined levels of 18S rRNA. Normalized mRNA levels were correlated with normalized AChE activity (Fig. 4B, 4C). For each construct, there exists a statistically significant linear correlation between the accumulation levels of AChE activity and those of *ACHE *transcripts when assayed either with the TEV 5'-probe (*hACHE-S*: r^2 ^= 0.73, *P = 0.0005*; *oACHE-S*: r^2 ^= 0.60, *P *<*0.0001*) or the VSP 3'-probe (*hACHE-S*: r^2 ^= 0.50, *P = 0.0104*; *oACHE-S*: r^2 ^= 0.49, *P *<*0.0001*). As expected, this suggests that for either type of recombinant gene, the accumulation of the enzyme product depends on the levels of its cognate mRNA, which vary among the different plant lines for each construct due to the positional effect of transgene integration [29,30], a phenomenon not unique to plants [19]. However, for both constructs, the distribution of transcript accumulation data is statistically indistinguishable for either the TEV 5'-probe (t test, *P *= 0.46, Insert in Fig. 4B) or the VSP 3'-probe (t test, *P *= 0.22, Insert in Fig. 4C). Furthermore, the quantitative real time RT-PCR assays directed, respectively, at the common 5' and 3' UTRs correlate well with each other (r^2 ^= 0.51, *P *<*0.0001*, data not shown). Thus, *oACHE-S *and *hACHE-S *transcripts accumulate to similar levels in the respective transgenic plants and it can be concluded that optimization of the *ACHE-S *sequence did not significantly increase transcript abundance, ruling out the latter as an explanation for the increased AChE activity in the *oACHE-S *plants. The explanation that neither transcription nor stability were affected seems more likely than the possibility that the optimization of the sequence resulted in simultaneous but opposite changes in transcription and stability (i.e. increased transcription with reduced transcript stability and *vice versa*).

### Optimization affects translation of the ACHE transcript

**Figure 5:**
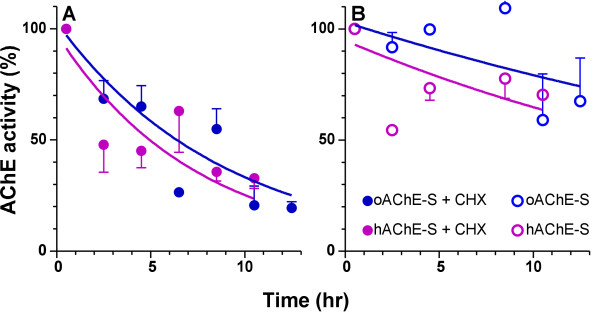
**Degradation of AChE proceeds at similar rates in plants expressing the optimized and non-optimized sequences**. Degradation was assayed by following the loss of enzymatic activity in leaf discs in the presence of the cytosolic protein synthesis inhibitor cycloheximide (A, +CHX) or in its absence (B). Plotted are means ± SEM of individual explant samples (*N *= 1–8). Non-linear regression analysis was performed using the Prism software with the resultant equations. oAChES + CHX: y = 103e^-0.11X ^(R^2 ^= 0.78); oAChES: y = 103e^-0.03X ^(r^2 ^= 0.36); hAChES + CHX: y = 98e^-0.14X ^(r^2 ^= 0.6123); hAChES: y = 95e^-0.04X ^(r^2 ^= 0.4463). The differences between the regression lines obtained in the presence of CHX are not significant (unpaired *t-*test, *P *= 0.195) while all other combinations are highly significant (*P *< 0.0001).

Having ruled out transcriptional and post-transcriptional events that may affect the accumulation level and the *bona fide *processing of the transcript of *hACHE-S*, we turned our attention to translational events. In our design of the optimized gene we took care not to introduce any amino acid changes and the mature protein products are expected to be identical. Nonetheless, our cloning procedure did necessitate minor changes in the N-terminus of the proteins, an R2G substitution in the *hACHE-S *sequence and insertion of A at the same position in the *oACHE-S *(Fig. 1). This region is part of the cleavable ER-targeting signal peptide in humans [18,31] and plants (SignalP3.0 server, [32,33] and Geyer et al, unpublished). To rule out the possibility that these small differences may effect the post-translational stability of the respective proteins, we incubated leaf explants from representative transgenic plants with or without cycloheximide to inhibit cytosolic proteinsynthesis (by blocking 80S ribosomes) and followed the degradation of the enzyme by assaying the residual AChE activity over a period of several hours (Fig. 5). The half-life time of the enzyme under these conditions is about 5 hours with no significant differences between *hACHE-S *or *oACHE-S *plants (*P *= 0.2, Fig. 5A). In the absence of cycloheximide (i.e. with, presumably, ongoing protein translation), a 20–30% loss of AChE is observed (Fig. 5B), which may be the result of the *ex-vivo *conditions of the experiments including the activation of wound-inducible proteases and depletion of the explants' energy resources. Interestingly, under these conditions that allow protein turnover, enzyme activity is lost somewhat (but significantly) slower in *oACHE-S *derived explants as compared to its loss in *hACHE-S *explants. This may suggest that AChE translated from human-sequence transcripts is replenished (resulting in greater net loss) less efficiently than AChE translated on plant-optimized transcripts.

Taken together, our results indicate that the higher levels of recombinant protein in the *oACHE-S *transgenic plants as compared to the *hACHE-S *plants are not due to transcriptional, post-transcriptional or to post-translational events. Rather, it appears that increased translation rates of the *oACHE-S *mRNA are responsible for the fact that AChE accumulates to higher levels in the *oACHE-S *plants.

Initiation is often considered to be the rate-limiting step for the translation process, and the regulation of mRNA sequences and secondary structures flanking the initiatory AUG are considered to play critical roles [34-36]. All of our constructs contained the same 5'-UTR (the TEV leader), but the 5'-proximal regions of the coding sequence were vastly different between the optimized and the human constructs in terms of their codon usage and GC content (Fig. 2). By *in silico *folding of the common 5'-UTR and the first 150 nucleotides of the two coding sequences (Mfold program [37]), it can be shown that in the six most-stable putative secondary-structures predicted for *hACHE-S*, the initiatory AUG is found within a relatively long and stable stretch of double helical RNA (ΔG° = 17.5 ± 0.0 kcal/mol, data not shown), while the equivalent region of *oACHE-S *is predicted to be at a base of a very short and considerably less-stable stem-loop structure (ΔG° = 6.3 ± 0.6 kcal/mol). It may therefore be speculated, based on these hypothetical structures, that the 5' proximal coding region plays an important role in controlling translation initiation.

**Figure 6:**
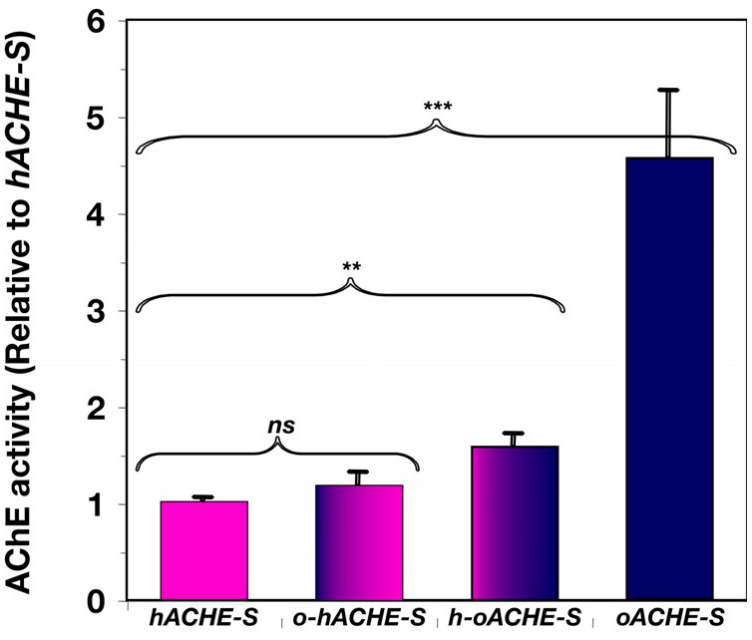
**Optimization of the 5'-proximal region of ACHE-S is critical but not sufficient for high expression levels**. The following constructs were transiently expressed by *A. tumefaciens*-infiltration of *N. benthamiana *leaves: *hACHE-S*, *o-hACHE-S, h-oACHE-S*, and *oACHE-S*. Averages of AChE activity of 6 replicates were normalized to that of *hACHE-S *to obtain the fold increase values (+ SEM) shown.

To test this hypothesis, we have swapped a 223 bp fragment of the 5' proximal region between the native and optimized constructs and created two chimeric sequences: *o-hACHE-S *and *h-oACHE-S *(Fig. 3A), which were transiently expressed in *N. benthamiana *leaves (Fig. 6). Expressing *h-oACHE-S*, which contained the native 5'-proximal sequence (with the rest optimized), resulted in a drastic and highly significant (*P *<*0.0001*) drop in the expression level when compared to the fully optimized sequence, but expression level was still modestly higher than for *hACHE-S*. In contrast, AChE accumulation levels driven by the construct *ohACHE-S *(in which only the 5'-proximal region of the sequence was optimized) were only marginally higher than, and statistically not different from, those obtained with hACHES (1.2 fold, P < 0.3, Fig. 6). Therefore, optimization of the 5'-coding region of the human *ACHE *gene, representing only about 10% of the entire sequence is necessary, but not sufficient, for enhanced expression of the gene in plants.

## Discussion

The history of molecular biology and its applications during recent decades is inseparably linked to the ability to express proteins in heterologous systems – i.e. the expression of a "target" protein from one species in "host" cells of another species [1]. From a biotechnology perspective, success in such efforts can be defined by "high-level" accumulation of the *bona fide *target in the host. This requires selecting the appropriate host species (and strain), defining its optimal growth conditions and/or optimizing genetic elements that control the expression and accumulation of the target. For example, expression in *Escherichia coli *of mammalian AChE, a glycoprotein, results in low yields of the unglycosylated, misfolded and destabilized protein [16], pointing to the need for a eukaryotic expression system. The importance of determining appropriate conditions is demonstrate by AChE of the electric ray, a cold-adapted fish, which, when expressed in mammalian cells, failed to accumulate if the cells were grown at 37°C, but could accumulate if the growth temperature was lowered [38]. Similarly, human AChE produced in *Pichia pastoris *was poorly secreted into the medium as compared to AChE from the snake *Bungarus fasciatus *[17,39], probably because of a transcription-attenuating GC rich region close to the 5'-end of the human coding sequence (Fig. 2) [31,39].

Our initial attempt at expressing human AChE in transgenic plants was modestly succesful, based on the definition suggested above: the enzymatic activity was indistinguishable from that of authentic human AChE, but the level of activity was fairly low [20]. An important factor responsible for the low observed activity in crude extracts was the presence of the anticholinesterase glycoalkaloid tomatine in the transgenic host – tomato plants [21]. However, low expression/accumulation levels of foreign proteins in transgenic plants have unfortunately been the norm rather than the exception, despite an obvious, and rather natural, bias in the literature to focus on the latter (for a comprehensive case study see Diehn et al [40]). Low yields of the recombinant protein product can be the result of limitations at every stage of gene expression, from transcription, through transcript processing and half-life, translation, post-translational modifications and turnover. Several strategies have been developed to overcome such limitations, including expression in plastids [41,42], use of virus-assisted expression systems [43-45], and optimizing the expression cassette to include strong promoters, appropriate 5'- and 3'-UTR, fusing targeting sequences, and conforming codon usage to that of plants [40,46]. Plastid expression is precluded because AChE is a glycoprotein and viral systems able to support the expression of >70-kDa proteins have only just become available for testing [45]. We therefore opted to focus on optimization of the gene sequence, which was synthesized *de novo *(Fig. 1, 3A), and was introduced into the low-alkaloid species *N. benthamiana *[47], lacking appreciable anticholinesterase activity (data not shown).

We present evidence for a substantial (>40 fold) increase in the accumulation of AChE activity in soluble protein extracts obtained from transgenic *N. benthamiana *plants expressing the full length S variant of human AChE as compared to our previously reported results concerning the expression of the AChE core domain in tomato plants (compare Fig. 3 and Fig 6 to Mor et al. [20] and Fletcher et al. [21]). In order to directly assess the effects of coding sequence optimization on the expression of the gene and the accumulation of its product, we compared the performance of the optimized gene construct (*oACHE-S*) to its native counterpart (*hACHE-S*) in *N. benthamiana *by transient expression assays (Fig. 3B) as well as in stable transformants (Fig. 3C). We demonstrate that optimization results in a 5 to 10 fold increase in the accumulation of the enzyme. Our results imply that a major contribution to the successful expression presented here is due to the optimization of the construct, although there were species- and isoform specific effects. For example, expression in tomato plants was considerably lower than in *N. benthamiana *and expression of the catalytic domain (constituting all but the 40-residue long C-terminal domain) was lower than that of AChE-S (unpublished observations). The latter may reflect the stabilizing effect of the carboxyl-terminal peptide of the S isoform [48]. Our results further establish that expression of AChE in *N. benthamiana *appears to have been marginally, if at all, limited by transcriptional, post-transcriptional, or post-translational events (Fig. 3, 4, 5, 6). It stands to reason, therefore, that enhanced translation of *oACHE-S *transcripts as compared to those of *hACHE-S *is responsible for the better performance of the latter construct.

Without dismissing other explanations, most previous reports suggested that limitations in expression of transgenes in plants are mainly due to unfavorable mRNA metabolism, more specifically to reduced synthesis, premature polyadenylation, aberrant splicing and reduced stability of transcripts of foreign gene in plant cells (reviewed by Diehn et al. [40]). Indeed, the case is quite compelling for at least one well-studied group of transgenes encoding the insecticidal δ-endotoxins of *Bacillus thuringiensis *(B.t.). When introduced into plant nuclear genomes, B.t. genes expressed poorly, and the dismal expression levels correlated well with low abundance of the full-length transcripts. A systematic and thorough investigation into the molecular mechanisms of low B.t. mRNA levels (and the consequential low levels of B.t. toxins) by Pamela Green and co-workers, led to their conclusion on the importance of "instability of mRNA as a cause of limited expression of foreign genes" in general [13]. B.t. genes, like many other eubacterial genes, are AT-rich and have multiple AU-rich destabilizing sequences and AU-rich plant polyadenylation signals in their mRNAs. All of these factors may contribute to rapid mRNA decay [13,40]. The eubacterial codon-usage bias towards AU-rich codons was also concluded to contribute to mRNA decay [13,40], although rare codons are clearly not sufficient to cause transcript degradation [49]. We, like others, observe good correlation between the transgene transcript levels and protein accumulation levels. Nevertheless optimization did not increase mature mRNA abundance (Fig. 4) and we have no evidence for aberrantly shorter mRNA species in plants that express the non-optimized *hAChE-S *construct. Furthermore, eliminating three potentially deleterious RNA sequences from *hACHE-S *did not result in improved performance, while introducing them into *oACHE-S *did not lead to reduced expression. Thus, we conclude that mRNA metabolism is unlikely to be key element in controlling expression of the *ACHE *transgene in plants.

What could be, then, the factors that limit the expression of the human *ACHE *gene in plants? In many cases of heterologous protein expression, the problem that "proteins are often difficult to express outside their original context," [1] can be overcome by changing codons to reflect frequencies observed in highly expressed proteins in a particular expression system. Codon usage bias is the primary factor in limiting heterologous gene expression in prokaryotes [50,51], chloroplasts [52], and mammalian cells [53,54]. Codon bias is associated with translational bias: highly expressed genes tend to utilize a limited set of codons and avoid the use of others, while codon preference positively correlates with their cognate tRNA content [1,55]. Rare codons, especially when clustered in the 5'-coding region (as can be seen in *hACHE-S*, Fig. 2), can lead to ribosome stalling, which acts to reduce elongation rates [2], and in some cases (but evidently not the case here) may in turn destabilize the mRNA [13].

Codon bias is inherently linked to a skew in the sequence's GC content. Many of the genes whose expression was attempted in plants, including the B.t. genes, are bacterial and highly AU-rich. Because AU-rich sequences destabilize mRNA in plants [13], the rule of thumb was to elevate the GC content as part of the optimization process. At 65%, the GC content of human *ACHE *is particularly high, and in specific areas, such as the 5'-proximal region, it is 75% GC (Fig. 2). At the molecular level, the high GC content of the 5'-proximal region may act to stabilize secondary mRNA structures, which in the context of mammalian cells may attenuate the transcription machinery [31,39]. While we do not observe differences in the overall steady state levels of the *ACHE *transcript accumulating in either *oACHE-S *or *h-ACHE-S *plants, secondary structures also affects translation initiation [56-58] and may also have a role in controlling the translation elongation rate [59]. By creating chimeric constructs consisting of optimized and native human sequences we were able to show thatthe GC-rich 5' membrane proximal region comprising about 10% of the gene had a pronounced inhibitory effect on the expression level from the otherwise fully optimized gene (*h-oACHE-S *construct, Fig. 6). Interestingly, the reciprocal swap represented in *o-hACHE-S *didn't significantly improve the expression when compared to *hACHE*S. This demonstrates that the optimization of the 5'-proximal region of the coding sequence, while critical for efficient translation, could not by itself account for the high level of AChE accumulation seen in *oACHE-S *expressing plants. Our results therefore suggest that in plants, both translation initiation and elongation are the most likely stages to be affected by sequence optimization of the *ACHE *gene. Our results also raise the interesting speculation that while in plants the high GC content of the gene may be a major obstacle for translation while in mammalian cells, normally growing at a much higher ambient temperature (typically 10°C to 20°C higher) the region provides for yet another potential checkpoint for the highly complex regulation of *ACHE *gene expression in mammals [60].

## Conclusion

The work presented here leads to the conclusion that human AChE accumulation in plants is limited at the translation stage and that this limitation is specifically linked to its highly biased codon usage and GC content. Changing the sequence to conform it to those that typical of highly expressed genes in plants results in increased translatability of the mRNA without affecting its accumulation or processing. The contributions of codon-optimization and GC-content adjustment to expression enhancement are difficult to be separately assessed, but it is clear that both are important. While the overall GC content should be adjusted to resemble the general base composition of highly expressed genes of the expression host, one should also pay particular attention to more localized GC-content skews, revealed by the type of analysis shown here (Fig. 6). Similarly, there is evidence to reject "across the board" replacement of synonymous codons to the most preferred one for each amino acid and in fact rare codons at certain strategic points along the sequence play an important role in functional protein synthesis whereby pausing at such rare codons allows proper folding of the already synthesized protein domains [61,62]. We are currently evaluating the relevance of this last point for the *ACHE *gene. Additional factors, such as subcellular targeting of the protein product and its subunit composition may affect the levels of its accumulation in the transgenic host. Indeed, we have preliminary evidence demonstrating that ER retention further increases the accumulation of some AChE variants (but not others) in stably transformed plants to about 1% of total soluble protein (Geyer, Fletcher and Mor, unpublished). Such a level of expression allowed us to efficiently purify the enzyme and we are currently testing its organophosphate scavenging potential in animal models.

## Methods

### Codon usage bias, GC content and mRNA folding analyses

Codon frequencies were obtained from the Codon Usage Database [63,64]. Codon usage bias was calculated according to Sharp and Li [55]. Briefly, the mRNA sequences encoding a group of highly expressed proteins from the model dicotyledonous plant *Arabidopsis thaliana *were selected to provide a reference for the codon usage bias. These included the small subunit 1B of RuBisCO, chlorophyll A/B binding protein 2, ribosomal protein L1 and L2, 40S ribosomal protein S2, S3 and S4, (accession nos. NM_123204, NM_102733, NM_202757, NM_201956 NM_115247, NM_115247 and NM_125228, respectively). The relative synonymous codon usage (RSCU = observed frequency/expected frequency assuming equal usage of synonymous codons for an amino acid), the relative codon adaptiveness (*w*, RSCU normalized to the most abundant synonymous codon for an amino acid) and the CAI values were calculated for the human *ACHE *gene sequence [31] according to Sharp and Li [55] (Fig. 2). Rare (or infrequently used) codons are arbitrarily defined here as having *w *values of less than 0.8. A moving CAI value (*n *= 50) was calculated for each codon. Similarly, the GC frequency was scored for each base as the average of a 100-base stretch centering on the particular base (Fig. 2). We have used the Mfold program [37] to predict mRNA secondary structures within the first 300 bases of the mRNA (centering on the initiatory AUG).

### Construction of a plant-expression vector for the human *ACHE-S *gene (*hACHE-S*)

Amplification of DNA was done using the Expand High Fidelity PCR kit (Roche), unless otherwise noted. Site directed mutagenesis was performed using the QuickChange kit (Stratagene). The gene encoding human AChE-S (*hACHE-S*) was PCR-amplified from pACHE-E6 [48] using the primers oTM001 and oTM005 (Table 2), cloned into pTOPOTA (Invitrogen) to yield pTM048. Site directed mutagenesis with primers oTM109 and oTM110 was performed to remove the single *Nco*I site within the gene to yield pTM180. An *NgoM*I*-Kpn*I fragment was used to replace the corresponding fragment in pTM034 [20] to yield pTM192 and its sequence was confirmed (Fig. 3). The expression cassette from pTM192, containing the *hACHE-S *coding region behind a cauliflower mosaic virus 35S (CaMV35S) promoter and the 5'UTR of tobacco etch virus (TEV leader) and in front of the 3'UTR of the soybean *vsp*B gene (VSP terminator), was cloned into the binary vector pGPTV-kan as described before [20] to yield pTM245 (Fig. 3). The binary vector pTM050 contained the *hACHE-S *gene with a single-base mutation.

**Table 2:** Oligonucleotides used in this study.

#	Name	5' Sequence 3'
1	TEVf	CAACATATACAAAACAAACGAATCTCAAG
2	TEVr	GCTATCGTTCGTAAATGGTGAAAA
3	TEVp	ATTCTACTTCTATTGCAGCAAT
4	oTM001	GATATCTGCAGCCATGGCTAGGCCCCCGC
5	oTM087	GTCTGCTACCAATATGTGGACACCC
6	oTM109	CTTTGCGGAGCCACCAATGGGACCCCGTCGC
7	oTM110	GCGACGGGGTCCCATTGGTGGCTCCGCAAAG
8	oTM089	CCCACCTTGTGGGCTGTCCTCCAGG
9	oTM090	CCTGGAGGACAGCCCACAAGGTGGG
10	oTM005	GGGTACCCGCCGGGGTCACAGGTCTG
11	oTM049	GAGTGTCTTAGGTGACTTACCCAC
12	oTM125	ATTTCCATGGGAAGGCCTCCACAATGTCTCCTCCACACTCCTTCCTTG
13	oTM127	CTCTTCCACAGGGCTGTGCTCCAATCTGGCGCGCCCAATGG
14	oTM128	GCCCACAGTGGCCCAGGGACCATTGGGCGCGCCAGATT
15	oTM126	TTCAGCAAGGACAATGAGTCTCTCATCTCCAGGGCTGAGTTCTTGGCTG
16	oTM129	CCAGTGGCACAATTGGCTGGAAGATTGGCTGCCCAAGGTGCC
17	oTM130	CTTCCAGCCAATTGTGCCACTGGGCACACCACATTGTGGTCTCCCAC
18	oTM131	TCCACCGCCACCACCGGTACCGAGCTCTCAGAGGTCTGAGCA
19	oTM047	AAGATAGGTGCTCAGACCTCTGAGAGCT
20	oTM085	GATACTCACAGAAAGAGGCTACTC
21	oTM086	CAGGATACGGGGAGCTAATGCAG
22	oTM095	GCCCAATGGACCCTGGGCC
23	oTM096	GGCCCAGGGTCCATTGGGC
24	oTM097	GTTCCCCAAGTaagtGACTTGGCTGC
25	oTM098	GCAGCCAAGTCacttACTTGGGGAAC
26	oTM099	CTGGAGCACAACAgtacGTTTCTTTGGACC
27	oTM100	GGTCCAAAGAAACgtacTGTTGTGCTCCAG
28	oTM101	CCCCCAATGGtCCCTGGGCC
29	oTM102	GGCCCAGGGaCCATTGGGGG
30	oTM103	GGTTCCCCAGGTttcaGACCTGGCAG
31	oTM104	CTGCCAGGTCtgaaACCTGGGGAACC
32	oTM105	GGGGCTCAGCAatatGTTAGTCTGGACC
33	oTM106	GGTCCAGACTAACatatTGCTGAGCCCC
34	oTM165	CCATGGGACCTAGGCGCTTTCTGC
35	oTM166	GCAGAAAGCGCCTAGGTCCCATGG
36	oTM167	CCAATGGGACCTAGGAGGTTTCTCCC
37	oTM168	GGGAGAAACCTCCTAGGTCCCATTGG
38	VSPf	GCTAGAGTTTGCTCCTATCTATATGTAATAAGG
39	VSPr	CATTAACAAACATAGCTAATGCTCCTATTT
40	VSPp	ATGCTGATATGCACTATT

### Construction of a plant-optimized *ACHE-S *gene (*oACHE-S*) and plant expression vector

The *oACHE-S *gene was assembled by the method of Stemmer et al. [65]. Briefly, 78 partially overlapping oligodeoxyribonucleotides, collectively encoding both strands of *oACHE-S *were synthesized (Integrated DNA Technologies, Inc., Coralville, IA), ranging 3560 bases in length. Exact lengths of the oligonucleotides were determined so that the overlap T_*m *_would be 60 ± 2°C. Oligonucleotides representing three sections of the gene (bases 1–785, 786–1333, 1334–1848, Fig. 1, 3A) were separately mixed and subjected to PCR amplification. Next, 1/20th of the PCR-amplified mixtures were subjected to a second round of PCR using flanking primers to yield three fragments for the three sections. The fragments were gel-purified, cloned into pTOPOTA (Invitrogen), to yield pTM052, pTM053 and pTM057. An *Asc*I-*Sac*I fragment of pTM053 was cloned into the respective sites of pTM052 to yield pTM075. An *Mfe*I-*Kpn*I fragment of pTM057 was then cloned into pTM075 to yield pTM079, containing all three *oACHE*-S fragments. The plasmid was sequenced and a singlebase mutation was corrected (sequence of *oACHE-S *was deposited with GenBank, accession no. DQ140345, Fig. 3). An *Nco*I-*Kpn*I fragment from pTM079 was used to replace the corresponding fragment in pTM034, yielding the intermediate vector pTM084, and the pGPTVkan based binary vector, pTM092 (Fig. 3).

### Construction of variants of *hACHE-S *and *oACHE-S *genes

Alterations of specific sequences in the *hACHE-S *and *oACHE-S *genes were introduced by site directed mutagenesis as follows (for the oligonucleotides used, see Table 2). The pea poly-A signal in pTM048 was mutated (Fig. 3) [25] using primers oTM101 and oTM102 to yield pTM152. The two 5'-intron splice signals present in pTM048 were sequentially mutated by using oTM103 and oTM104 to disrupt one signal, yielding pTM153, and then using oTM105 with oTM106 to disrupt the other, yielding pTM161. Similarly, the two 5'-intron splice signals were mutated in pTM152 to yield pTM157, resulting in a *hACHE-S *variant lacking all three potentially deleterious sequences. We then replaced the *NgoM*I-*Kpn*I fragment in pTM034 with the corresponding fragment in pTM152, pTM161, and pTM157, obtaining pTM160, pTM162, and pTM158, respectively. Following sequence confirmation, the expression cassette was cloned into the binary vector, pGPTV-kan, as described above to yield *hACHE-S *constructs, pTM163 (minus poly-A), pTM166 (minus 5'intron splice sites), and pTM159 (minus poly-A and 5'-intron splice sites).

The three potentially deleterious sequences were introduced back into the *oACHE-S *sequence in an analogous way. The mutagenesis oligonucleotides oTM095, oTM096 were used to recreate the poly-A signal in pTM079 to give pTM189. An *Nco*I-*Sac*I fragment was used to replace the corresponding fragment in pTM034 to yield pTM194 and its sequence was confirmed. The two 5'-intron splice signals were introduced into the sequence of pTM053 and pTM057 with oligonucleotides oTM097, oTM098, oTM099 and oTM100 yielding pTM169 and pTM148 respectively. The plasmids were sequenced and the full-length *oACHE-S *coding sequence (with the splicing signals) was reassembled from an *Asc*I-*Mfe*I fragment of pTM169, an *Mfe*I-*Sac*I fragment of pTM148, and the *Sac*I-*Asc*I backbone of pTM084 by a triple-ligation cloning step, yielding pTM172. Reintroduction of all three potentially deleterious signals was similarly carried out yielding pTM151. Following sequence confirmation, the expression cassettes from pTM194, pTM172, and pTM151 were cloned into the binary vector pGPTV-kan, as described above to yield *oACHE-S *construct variants, pTM196 (+poly-A), pTM193 (+5'-intron splice sites), and pTM154 (+poly-A, +5'-intron splice sites).

### Construction of mosaics of *hACHE-S *and *oACHE-S *genes

An *Avr*II restriction site was introduced into the *hACHE-S *plasmid, pTM048, and the *oACHE-S *plasmid, pTM084 (with the respective primer pairs, oTM165 and oTM166, and oTM167 and oTM168, see Table 2), yielding pTM241 and pTM235, respectively. Following sequence verification, a *Xho*I*-Sac*I fragment from pTM235 was cloned into pTM084 to yield pTM238, while an *NgoM*I*-Kpn*I fragment from pTM241 was used to replace the corresponding fragment in pTM034 to yield, pTM242 (Fig. 3A). The *Hind*III*-Avr*II fragment from pTM238 was used to replace the corresponding fragment from pTM238, yielding pTM252. Similarly, an *Avr*II*-Sac*I fragment from pTM238 was used to replace the corresponding fragment from pTM242, to yield pTM253. The expression cassettes from pTM252 and pTM253, complete with the aforementioned control elements of pTM034 were then cloned into the binary vector, pGPTV-kan, as described above, to yield pTM256 (*o-hACHE-S*) and pTM257 (*h-oACHE-S*), respectively.

### Stable and transient transformation of *Nicotiana benthamiana*

Binary vectors containing genes of interest were electroporated into *Agrobacterium tumefaciens *LBA4404 and were used in the subsequent transformation of *N. benthamiana *explants (>50 each) as described before [66]. Kanamycin-resistant plants were regenerated as described [66]. *Agrobacterium*-mediated transient expression assays were conducted essentially as described before [67,68]. Briefly, *A. tumefaciens *cells harboring the appropriate expression vectors were grown for 24 hours, harvested by centrifugation and resuspended to culture density of 0.4 OD_600 _in 10 mM MES (pH 5.5) and 200 μM acetosyringone. Test constructs were co-expressed with the silencing suppressor protein, p19 [68]. To this end, *A. tumefaciens *cells carrying each test plasmid were mixed (1:1) with *A. tumefaciens *cells carrying a CaMV 35S promoter-driven p19 expression vector (kind gift of Dr. Hugh Mason). After nicking the abaxial leaf epidermis with a needle, the bacterial suspension was infiltrated into the leaf through a blunt tip of a 1 ml syringe. Five days post-infection, the infiltrated-leaf tissue was harvested and homogenized as described below.

### Biochemical and Molecular analyses

Genomic PCR was preformed on 0.1 μg total DNA isolated from kanamycin resistant plants (DNeasy kit, Qiagen) using either primers oTM087 and oTM090 or primers oTM126 and oTM047 to amplify *hACHE-S *or *oACHE-S *respectively (Table 1). RNA blot analysis on total leaf RNA (RNAqueous kit, Ambion) was done as described before [20] using a digoxigenin-labeled probes that were synthesized with primers oTM087 and oTM090 and pTM079 or pTM048 as templates.

For real-time RT-PCR analysis, total cellular RNA was prepared by homogenizing 100 μg of leaf tissue in a FastRNA-Pro-Green tube containing lysis matrix (QBiogene) in the presence of 800 μL Lysis/Binding solution (Ambion) using a FastPrep machine (Qbiogene). Lysates were then processed according to manufacturer's instructions for RNAqueous kit with the plant RNA Isolation Aid (Ambion). Residual genomic DNA was removed using the DNAfree system (Ambion). Yield and purity were determined spectrophotometerically and then RNA samples were reverse transcribed (RETROscript, Ambion) using random priming. To assess the quality of the cDNA, samples were screened by PCR with the primers oTM085 and oTM086 specific to the *N. benthamiana *actin gene (AY179605) and only those samples producing the expected 0.36 kb fragment were set aside for further processing (data not shown). Real-Time PCR was done as follows using an ABI Prism 7900HT sequence detection system (Applied Biosystems). Amplification of RNA samples (18 ng) was performed in triplicates in a 384-well plate with primers specific to the common 5'-UTR TEV leader and 3'UTR VSP terminator sequences (TEVf, TEVr, VSPf and VSPr, see Table 2) and custom-made TaqMan^® ^FAM/MGB probes (TEVp and VSPp, Applied Biosystems). As an endogenous reference served 18S rRNA which was amplified using the TaqMan^® ^eukaryotic 18S rRNA primers and FAM/MGB Probe (Applied Biosystem). Transcript levels were quantitated using two separate standard curves for the 18S rRNA reference and the *ACHE *transcript (using sample *oACHE*-S-24B) and normalized (*ACHE *value/18S value).

RT-PCR was also used to test for the presence of shorter, aberrantly spliced transcripts using cDNA samples (50 ng in a 25 μl reaction) and oTM089 and oTM049 as primers (35 cycles). In order to amplify even very rare templates, 1 μl samples of the above PCR reactions (total new reaction volume was 100 μl) were subjected to a second 35-cycle round of PCR, and the products were resolved by agraose gel electrophoresis.

To assess the ex-vivo stability of plant-expressed AChE, leaf discs (7 mM) from seedlings were floated, abaxial side up, in diH_2_O or in diH_2_O supplemented with 1 mM cyclohexamide [Sigma, 69]. At the indicated time points, leaf discs were sampled (n = 10), blotted dry, snap frozen in liquid N_2 _and stored at -80°C until further analysis.

Leaf samples were homogenized in ice-cold extraction buffer (1 M NaCl, 25 mM Tris, 0.1 mM EDTA, 10 μg/ml leupeptin (Sigma), and 0.5% Triton X-100, pH 7.4, 3 ml per 1 g tissue) using ceramic beads in a FastPrep machine. Ellman cholinesterase assays using acetylthiocholine (ATCh, Sigma) as substrate in the presence or absence of the selective AChE inhibitor 1,5-bis(allyldimethylammoniumphenyl)pentan-3 one dibromide (BW, 10 μM, Sigma) were done as described [20]. BW-inhibitable ATCh hydrolysis rates were monitored at 405 nm for 30 min with a SpectraMax 340PC spectrophotometer, (Molecular Devices) and specific activity has been reported in mU/mg protein (AChE unit definition here is, 1 μmol ATCh hydrolyzed in 1 min at 25°C). Total soluble protein was determined as described before [20].

### Statistical analyses

Student t test for two-group comparisons was performed with GraphPad Prism 4.0c for Macintosh (GraphPad Software, San Diego California USA). Multi-group comparisons were done using the non parametric exact permutation test with the PGD program [70] and *P *values were computed based on 10 repeat blocks of 1,000 permutations each.

## Abbreviations

AChE, acetylcholinesterase; AChE-S, synaptic isoform of AChE; ATCh, acetylthiocholine; CAI, codon adaptiveness index; CaMV35S, cauliflower mosaic virus 35S promoter; *hACHE-S*, sequence of the human gene encoding AChE-S; *oACHE-S*, plant expression optimized gene; OPs, organophosphates; RSCU, relative synonymous codon usage; RuBisCO, ribulose bisphosphate carboxylase; TEV, tobacco etch virus; VSP, vegetative storage protein.

## Authors' contributions

SPF and BCG designed experiments, carried out cloning, plant transformation and molecular genetic analyses and drafted sections of the manuscript. TAG performed the statistical analyses. MJL substantially contributed to plant transformation and transformant screening. HS contributed to writing the manuscript. TSM conceived the study, directed its design and coordination and wrote the manuscript. All authors read and approved the final manuscript.
